# Attentional deployment impacts neural response to regret

**DOI:** 10.1038/srep41374

**Published:** 2017-02-01

**Authors:** Zhiyuan Liu, Lin Li, Li Zheng, Min Xu, Fanzhi Anita Zhou, Xiuyan Guo

**Affiliations:** 1 Shanghai Key Laboratory of Magnetic Resonance and Department of Physics, East China Normal University, Shanghai, SH, China; 2 Department of Psychology, School of Psychology and Cognitive Science, East China Normal University, Shanghai, SH, China; 3 Key Laboratory of Brain Functional Genomics, Ministry of Education, Shanghai Key Laboratory of Brain Functional Genomics, School of Psychology and Cognitive Science, East China Normal University, Shanghai, SH, China; 4 Zhejiang Yuying Education Group, Hangzhou,China

## Abstract

Regret results from comparing non-optimal outcomes containing both gain and loss parts to better alternatives during decision-making. The present fMRI study aimed to explore whether levels of regret could change when gain or loss part of a non-optimal outcome was focused during a sequential risk taking task, i.e. the effect of an important emotional regulation strategy named as attentional deployment on regret. Participants were asked to open a series of boxes consecutively and decided when to stop. Each box contained a reward, except for one containing a devil to zero one’s gain in the trial. After participants stopped, both collected gains and missed chances were revealed. Besides, participants were induced to focus on collected gains (GF) or missed chances (MF), by highlighting different parts of the outcome with red squares. Behaviorally, participants rated less regret to their decisions in GF than MF context. Moreover, participants tended to be less risk-taking after GF context relative to MF. At the neural level, bilateral straitum showed increased activations in both optimal outcome and GF context. However, right MFG and IPL only showed stronger activation in GF than MF context. Additionally, pgACC and mPFC activations were found only in optimal outcome.

Individuals make thousands of decisions daily aiming to approach positive outcomes and avoid negative outcomes. An optimal outcome would surely introduce positive emotions such as relief^1^,^2^. In most cases, however, outcomes of decisions are not optimal ones. Generally a typical outcome tends to be non-optimal and seems like a mixture of both gains and missed chances. Numerous previous researches indicated individuals tended to compare non-optimal outcomes of their choices to some better alternatives, after which they could experience negative emotion of regret^3^,^4^,^5^. As illustrated by the opening quotation, the emotion of regret might be related to individuals’ sensitivity to missed chances than gains^6^,^7^. However, nearly everything could be perceived in two different ways. This is elegantly captured by the frequently cited idiom “*Is the glass half full or half empty?*”. In the case of encountering a non-optimal outcome, this merely means that the objective emotional experience of an individual could change according to which part of the outcome has been focused on (focusing on gains or missed chances). Thus, we would investigate whether focusing on gains could repress the emotion of regret compare to focusing on missed chances.

In psychological literature, focusing on ‘good’ or ‘bad’ parts is a popular manipulation of the focus of attention, which serves as an important emotional regulation strategy named as attentional deployment^8^,^9^,^10^. Attentional deployment tends to be a bottom-up strategy and requires less cognitive resource^10^,^11^. In previous research, attentional deployment was usually manipulated by directing participants focused attention towards arousing (eg. negative) or non-arousing (eg. neutral) parts of unpleasant pictures^10^,^12^. Behaviorally, these previous research revealed that participants felt significantly less negative emotion after focusing their attention on non-arousing parts of the unpleasant pictures relative to arousing parts. Accordingly, neuroimaging results revealed that the non-arousing focus condition was associated with increased activity in frontal regions like middle frontal gyrus (MFG), compared to the arousing focus condition^10^. As an explanation to the neuroimaging finding mentioned above, some argued that negative information had the capacity to automatically capture attention^13^, so that increased activation in MFG might reflect additional effort and cognitive control required to direct and maintain focus in a relatively non-arousing compared to an arousing region of the same unpleasant picture^10^.

In the present study, we adopt a similar method to induce attentional deployment during the outcome phase of a sequential risk taking task, which has been used to prompt the emotion of regret^14^,^15^. Specifically, in the sequential risk taking task, participants were asked to open a series of boxes consecutively and decide when to stop. All except for one box contained a reward (gold); while the only exceptional contained an adverse stimulus (devil) to zero one’s gain in the trial. When participants decided to stop, the position of devil was shown, indicating values of their collected gains and missed chances. Importantly, we manipulated attentional deployment by highlighting missed chances or collected gains with squares, which induced participants focusing on either the good part (their collected gains) or the bad part (their missed chances) of the outcome. And then participants were asked to rate the emotional states of regret or relief for their decisions.

We hypothesize that compared to missed chances, focusing on collected gains could repress the emotion of regret. So behaviorally we predict that participants might feel better (rate more relief and less regret) after focusing on collected gains relative to missed chances. At the neural level, previous studies have identified the neural structure of striatum related with reward processing^16^,^17^,^18^. Recently, by using the sequential risk taking task, some research showed that striatum was participating in regret processing, whose activation decreased along with increasing regret level^14^,^15^. Moreover, previous research also found that pregenual anterior cingulate cortex (pgACC) and medial prefrontal cortex (mPFC) were involved in reward processing^15^,^19^,^20^,^21^,^22^. Therefore, firstly, we predict that striatum, pgACC and mPFC might show shronger activations when participants focused on collected gains compared to missed chances. Secondly, in line with previous research^10^, we predict that focusing on collected gains compared to missed chances might be associated with increased activations in frontal regions, like MFG.

## Results

### Behavioral results

Firstly, we compared the number of collected gains in both GF (focusing on collected gains) and MF (focusing on missed chances) contexts and the number of missed chances in both GF and MF contexts. Paired-t test revealed no significant difference of collected gains and missed chances (*ts* < .6, *ps* > .5) between GF and MF contexts.

**Figure 1:**
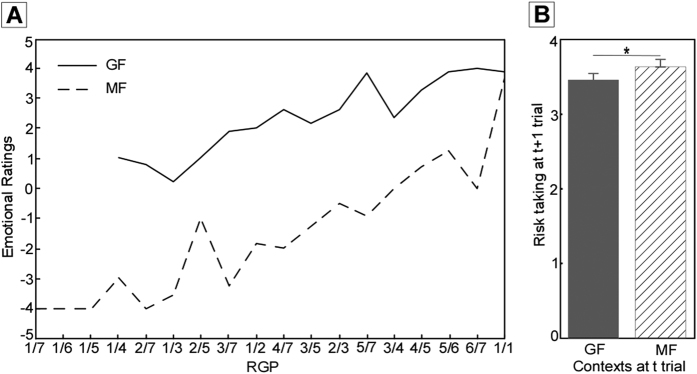
Behavioral results. (**A**) The relationship between participants’ emotional ratings and RGP in GF and MF contexts. Results revealed that participants experienced less regret (i.e. more relief) if they focused on collected gains relative to missed chances. (**B**) Behavioral changes after participants focused on collected gains or missed chances in gain_gain condition. Paired-t test revealed that participants tended to open more boxes at t + 1 trial after they focused on missed chances compare with collected gains at t trial (t(17) = 2.20, p < .05).

The outcome of each trial might be one of the following three: (i) Non-optimum (Nopt), in which participants did not unpack the devil though missed some chances, (ii) Optimum (Opt), in which participants did not unpacked the devil and got the largest possible gain (zero missed chances), (iii) Devil, in which participants unpacked the devil and lost the golds collected in that trial. To investigate how attentional deployment affected the emotion of regret, The 2 (Context: GF vs. MF) × 2 (Outcome: Nopt VS. Opt) repeated measures analysis of variance (ANOVA) on emotional ratings revealed significant main effects of Context and Outcome (both *Fs* < 453.9, both *ps* < .001), indicating higher emotional ratings in the GF context than in the MF context. A significant interaction between Context and Outcome was also found (*F(1,17)* = 79.9, *p* < .001). Paired t-tests revealed that emotional ratings for Nopt outcome in GF context were significant higher than those in MF context (*t(17)* = 10.5, *p* < .001). However, emotional ratings for Opt outcome showed no significant difference between GF context and MF context (*t(17)* = 1.8, *p* > .05). Moreover, we introduced a combined index, called real gain-percentage (RGP), which was defined as the ratio of the collected gain and the largest possible gain (that is, the total number of boxes before the devil, or the sum of the obtained gain and the missed chance) in a given trial^15^. Then the relationship between participants’ emotional ratings and RGP in both GF and MF contexts were described (Fig. 1A). This showed that emotional ratings were higher in GF context than in MF context in the case of same RGP.

In addition, to investigate the behavioral changes after participants focused on collected gains or missed chances, we restricted the analysis in Gain_Gain condition (trials in which participants did not unpack the devil in both the current and the next trials)^15^. In such condition, we compared the the number of opened boxes in the next trial (t + 1 trial) after participants focused on collected gains or missed chances in the current trial (t trial). Paired-t test revealed that participants tended to open more boxes after focusing on missed chances compare to collected gains (*t(17)* = 2.20, *p* < .05) (Fig. 1B).

### fMRI results

#### ROI analyses

**Figure 2:**
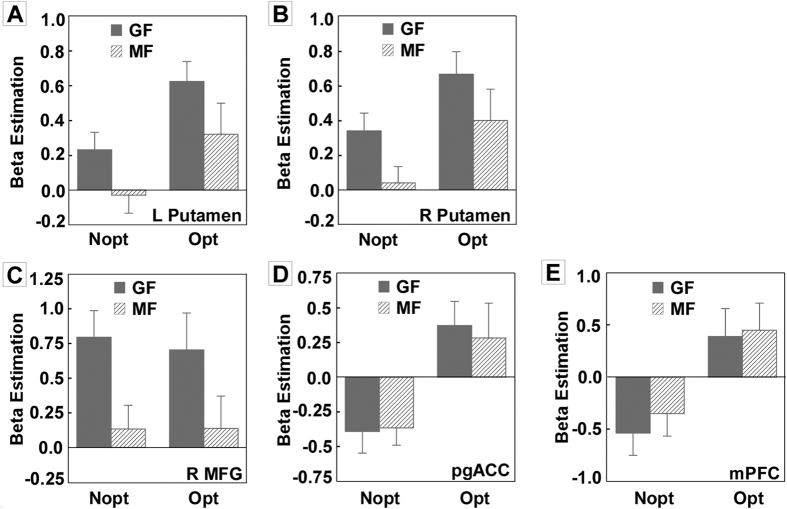
ROI analyses. The 2 (Outcome: Opt vs. Nopt) ×2 (Context: GF vs. MF) repeated measures analysis of variance (ANOVA) was used. The activations of left putamen and right putamen showed significant main effects of Context and Outcome (Fs > 20.76, ps < .05) (**A** and **B**). Moreover, the activation of right MFG only showed significant main effect of Context (F(1,17) = 23.37, p < .01), suggesting stronger right MFG activation for GF context than MF context (**C**). Contrary to the activation pattern of right MFG, activations of pgACC and mPFC only showed significant main effect of Outcome instead of Context, indicating stronger pgACC and mPFC activations for Opt outcome than Nopt outcome (Fs > 25.74, ps < .01) (**D** and **E**).

We extracted parameter estimates of signal intensity from each of the functionally defined ROIs (left putamen, right putamen, right MFG, pgACC and mPFC). Then the 2 (Outcome: Opt vs. Nopt) ×2 (Context: GF vs. MF) repeated measures analysis of variance (ANOVA) on parameter estimates of signal intensity was used (Fig. 2). The activations of left putamen and right putamen showed significant main effects of Context and Outcome (*Fs* > 20.76, *ps* < .05) (Fig. 2A and B). The result indicated greater bilateral putamen activations for GF context than MF context, and for Opt outcome than Nopt outcome. Moreover, the activation of right MFG only showed significant main effect of Context (*F(1,17)* = 23.37, *p* < .01), suggesting stronger right MFG activation for GF context than MF context (Fig. 2C). Contrary to the activation pattern of right MFG, activations of pgACC and mPFC only showed significant main effect of Outcome instead of Context, indicating stronger pgACC and mPFC activations for Opt outcome than Nopt outcome (*Fs* > 25.74, *ps* < .01) (Fig. 2D and E). For all ROIs, no significant interaction was found.

#### Whole-brain analyses

**Figure 3:**
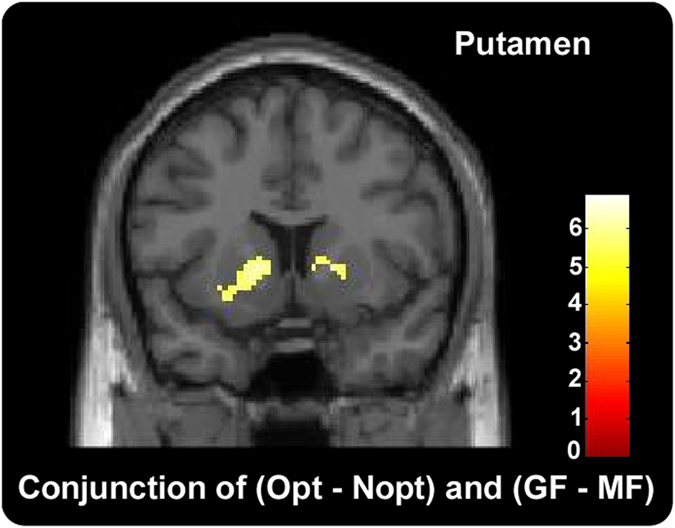
Conjunction analyses of (Opt - Nopt) and (GF - MF) contrasts. The result showed that common brain regions of Left putamen, right putamen and so on were activated by both (Opt–Nopt) and (GF–MF) contrasts.

Whole-brain analyses were conducted to verify results in ROI analyses. Firstly, a conjunction analysis using the conjunction null hypothesis^23^ was conducted to explore common brain regions activated by (Opt–Nopt) and (GF–MF) contrasts. The result revealed that left putamen (MNI −16 14–2), right putamen (MNI 18 10 2) and pgACC (MNI 8 36 12 and −6 28 16) were activated (Fig. 3 and Table 1).

**Figure 4:**
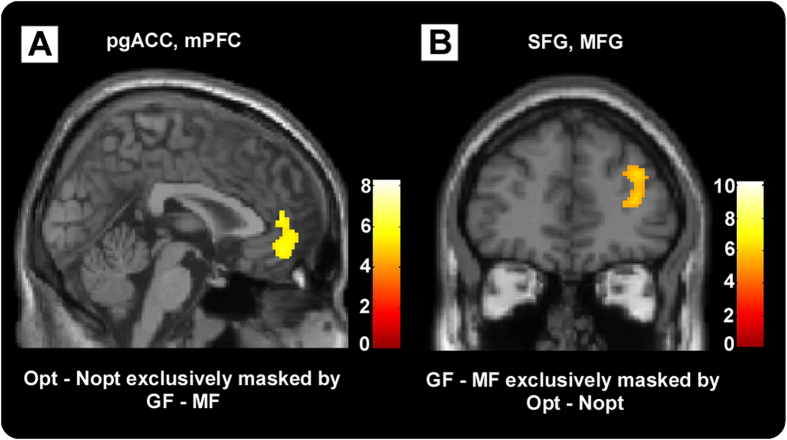
(**A**) (Opt - Nopt) contrast exclusively masked by (GF - MF) contrast. The result showed mPFC and pgACC and so on were activated only in the (Opt - Nopt) contrast. (**B**) (GF - MF) contrast exclusively masked by (Opt - Nopt) contrast. Right MFG, right SFG and so on were activated specifially in the (GF - MF) contrast.

Then, to investigate the difference between (Opt–Nopt) and (GF–MF) contrasts, two contrasts were masked by each other at a threshold of P < 0.05 (note that a more liberal threshold of the exclusive mask reflects a more conservative approach^24^). The result showed mPFC (MNI −4 54 2) and pgACC (MNI 8 46 6) were activated only in the (Opt - Nopt) contrast (Fig. 4A, Table 2). Whereas, right MFG (MNI 36 52 12), right Inferior parietal lobule (IPL, MNI 34–50 50) and right superior frontal gyrus (SFG, MNI 26 8 58) were activated specifically in the (GF - MF) contrast (Fig. 4B, Table 3).

Moreover, the interaction between Outcome (Opt vs. Nopt) and Context (GF vs. MF) did not show any significant activation, even at a liberal threshold (voxel-level *p* < .001, uncorrected).

In addition, bilateral putamen (left putamen, MNI −18 10 0; right putamen, MNI 20 12 2) and left pallidum (MNI −18–2 6) were significant in the Nopt - Devil contrast. The reverse contrast did not show suprathreshold activation (Table 4). Such results were accordant to our previous findings with same contrast using a similar paradigm.

Parametric analyses were also preformed to assess how brain activities were modulated by RGP in trials where participants did not unpack the devil. No regions survived.

## Discussion

The current study employed a modified sequential risk taking task to investigate whether focusing on gains could repress the emotion of regret on both behavioral and neural levels compare to focusing on missed chances. Behaviorally, consistent with our prediction, the results showed participants felt less regret (more relief) if they focused on collected gains relative to missed chances. Moreover, the results revealed that participants took less risks after focusing on collected gains compared to missed chances. At the neural level, greater activities within bilateral striatum were observed when participants focused on collected gains or encountering with optimal outcome. Besides, right MFG and IPL showed greater activation when participants focused on collected gains relative to missed chances, while activities in both pgACC and mPFC were greater in optimal outcome relative to non-optimal outcome.

In line with our hypothesis, participants felt less regret (more relief) after they focused on collected gains relative to missed chances, indicating focusing on gains could repress the emotion of regret on behavioral level. Studies on emotion regulation have postulated that attentional deployment played a critical role in mood and emotion regulation^8^,^25^. For instance, Ferri *et al*.^10^ revealed participants felt significantly less negative emotion after focusing their attention on non-arousing part of unpleasant images. In accordance with previous research, the current study suggested that attentional deployment was also an effective strategy in the regulation of regret. Additionally, our results showed participants tended to take more risks after focusing on missed chances. Such results might suggest that after focusing on missed chances of the preceding trial, participants were more likely to be risk-taking (to open more boxes), presumably because of their motivation to avoid missed chances in the followed trial.

In the current study, significant striatum activation was found in both Opt - Nopt and GF - MF contrasts. Specifically, previous studies that employed similar sequential risk taking tasks have also identified striatum activity in the Opt - Nopt contrast^14^,^15^. Moreover, abundant studies showed the brain region of striatum involved in reward processing^17^,^26^,^27^,^28^,^29^. The greater striatum activation in optimal outcome relative to non-optimal outcome in the current study also confirmed the reward-related role of the striatum. Besides, in line with previous finding that increased regret was found along with decreased activation of striatum^14^,^15^, in the current study, stronger striatum activation was found when participants were focusing on collected gains than missed chances. Such neural results were consistent with our behavioral results that participants experienced less regret (more relief) when they focused on collected gains relative to missed chances. Together, our results suggested that focusing on gains could repress the emotion of regret on both behavioral and neural levels compared to focusing on missed chances.

Importantly, stronger frontal, as well as parietal regions, like right MFG, right SFG and right IPL activations were found when participants focusing on collected gains relative to missed chances. Such results were consistent with previous finding that MFG, SFG and IPL were more active when participants had to maintain focus in a relatively non-arousing compared to an arousing region of the same picture^10^. Many studies showed that people were more sensitivity to negative information than positive information when making a decision^6^,^7^. On account that negative information had the capacity to automatically capture attention^13^, increased activation in brain regions of MFG, SFG and IPL might reflect additional effort required to direct and maintain attention to collected gains. Prefrontal and parietal regions are considered essential for cognitive control during effortful tasks^30^, and fronto-parietal networks are known to underlie visuospatial attention and visual control^31^.

Our findings on pgACC and mPFC did not completely fit the hypothesis. Stronger pgACC and mPFC activations were found only in the contrast between optimal and non-optimal outcome, but not in the contrast between GF and MF. We suggested that increased activations in pgACC and mPFC along with optimal output reflect their roles in a ‘reward system’ which has been repeatedly identified during decisions involving rewards^15^,^19^,^20^,^21^,^22^. The current finding on pgACC and mPFC was also in line with our own previous findings that these regions were associated with optimal outcome. Except for the reward related role of mPFC and pgACC, previous research has also identified them as areas that responded to error feedback^32^,^33^,^34^,^35^. The error feedback function might serve as a better explanation to the findings on mPFC and pgACC in the current study. As mentioned above, the optimal outcome could be considered as the unexpected stimulus. So, in general, prediction error would be greater in trials with optimal outcomes, relative to trials with non-optimal outcomes. On the other hand, any given outcome was fixed by itself, regardless of manipulations on the focus of attention. As a result, the magnitude of error feedback, if there is any, in GF context might not differ from that in MF context. Hence the stronger pgACC and mPFC in optimal outcome might suggested that pgACC and mPFC were associated with not only reward processing but also error feedback processing.

## Conclusion

The current study suggested that focusing on gains could repress the emotion of regret on both behavioral and neural levels compare to focusing on missed chances. Focusing on collected gains of an outcome, relative to missed chances, was associated with reduced emotion of regret, increased bilateral striatum, right MFG and right IPL activation. Moreover, stronger striatum activation was found in optimal outcome relative to non-optimal outcome. The stronger striatum activation in both optimal outcome and focusing on collected gains context suggested the reward related role of striatum. Increased activation in brain regions of right MFG and right IPL when participants focused on collected gains might reflect additional effort required to direct and maintain attention to collected gains.

## Methods

### Participants

Eighteen right-handed participants (ten females, aged from 19 to 28, *M* = 23.94, *SD* = 2.39) from the university community with normal or corrected-to-normal vision participated in this experiment. None of the participants had abnormal neurological history. All of them gave informed consent before scanning. This study was approved by the Ethical Committee of East China Normal University. The methods were carried out in accordance to approved guidelines and regulations.

### Procedure

Before scanning, participants were told that they would play a sequential risk taking task while undergoing fMRI scanning. Participants were also informed that the payment for their participation would be affected by their gains from the task. The actual payment was calculated by subtotaling gains of 30 randomly chosen gain trials at the end of the task.

**Figure 5:**
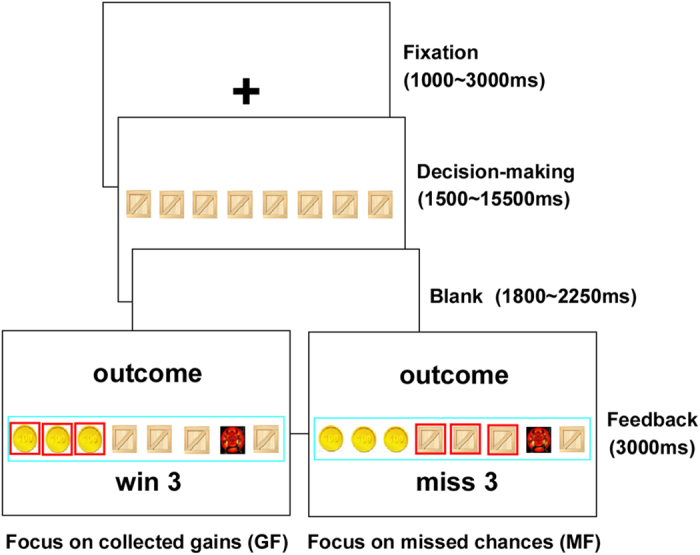
Two possible conditions are displayed when participants play the task undergoing fMRI scanning. Participants decide to stop after collecting three gold coins. The outcome revealed that they collected three gold coins and missed three chances (i.e. non-optimal outcome) in the current trial. The cue (red squares) might induce participants focus on collected gains or missed chances. Focusing on collected gains (GF) was induced by highlighting each collected gain with a red square, and a text indicating three gold coins they have collected. Moreover, focusing on missed chances (MF) was induced by highlighting each missed chance with a red square, and a text indicating three chances they have missed.

Participants completed 90 trials in the scanner. On each trial, they were shown an array of eight boxes on the computer screen, where seven boxes contained gains (golds) and one box contained a loss (devil). Devil was set in the first box for 3 trials. There were 48 trials in which devil was set from the second box to the fifth box, and it was set in each box for 12 trials. For the remained 39 trials, devil was set in the sixth, seventh, or eighth box, in equal number of trials (13 trials for each position). The position of the devil was set randomly on each trial leading to no significant autocorrelation. Boxes were opened from left to right. At any stage, participants had 2000 ms to either open the next box or stop and collect the gains acquired so far in that trial by key-press. Opening the box with the devil ended the current trial and all gains from that trial were lost. A jittered interval (ranging from 1800 to 2250 ms) was presented after the participant decided to stop, or after the unpacking of the devil. Then the outcome was presented for 3000 ms and highlighted on the screen by a cyan square (in the case of stopping and collecting the gains) or a red square (in the case of unpacking the devil and losing the gains in that trial). The outcome screen also revealed the actual position of devil, thus informing participants about how many golds they’d collected and how many chances they’d missed at the same time. The outcome of each trial might be one of the following three: (i) Non-optimum, in which participants did not unpack the devil though missed some chances, (ii) Optimum, in which participants did not unpacked the devil and got the largest possible gain (zero missed chances), (iii) Devil, in which participants unpacked the devil and lost the golds collected in that trial. Importantly, we manipulated attentional deployment by the use of cues (red squares) and text feedback, inducing participants to be focusing on collected gains or missed chances. Focusing on collected gains (GF) was induced by highlighting each collected gain with a red square, and a text indicating the total number of gains. Meanwhile, focusing on missed chances (MF) was induced by highlighting each missed chance with a red square, and a text indicating total number of missed chances. Finally, an additional jittered inter-trial interval (ranging from 1500 ms to 15500 ms) was introduced. Figure 5 displays two of the possible conditions for a trial.

After scanning, participants were presented with their own choices in every trial from the task completed inside the scanner. Specifically, for each trial, participants watched the outcome with cue (identical to the feedback screen participants watched in the scanner) and were asked to rate how they felt for this trial on a 9-point scale from extreme regret (defined as −4) to extreme relief (defined as 4).

### fMRI data acquisition

Scanning was carried out on a 3 T Siemens Trio system at the Functional MRI Lab (East China Normal University, Shanghai). For functional images, 35 slices were acquired using a gradient-echo echo-planar imaging (EPI) sequence (TR = 2200 ms, TE = 30 ms, FOV 10 = 220 mm, matrix size = 64 × 64, slice thickness = 3 mm, gap = 0.3 mm). Before the functional run, a high-resolution structural image was acquired using a T1-weighted, multiplanar reconstruction (MPR) sequence (TR = 1900 ms, TE = 3.42 ms, 192 slices, slice thickness = 1 mm, FOV = 256 mm, matrix size = 256 × 256).

Data pre-processing and statistical analyses were performed with Statistical Parametric Mapping (SPM12, Wellcome Department of Cognitive Neurology, London). The functional images were corrected for the delay in slice acquisition and were realigned to the first image to correct for interscan head movements. The individual T1-weighted, 3D structural image was co-registered to the mean EPI image generated after realignment. The co-registered structural image was then segmented into gray matter (GM), white matter (WM) and cerebrospinal fluid (CSF) using a unified segmentation algorithm^36^. The functional images after slice timing and realignment procedures were spatially normalized to the Montreal Neurological Institute (MNI) space (resampled to 2*2*2 mm^3^) using the normalization parameters estimated during unified segmentation and then spatially smoothed with a Gaussian kernel of 8 mm full-width half-maximum (FWHM).

### fMRI data analyses

A two-level random effects approach utilizing the general linear model as implemented in SPM12 was used for statistical analyses. At the first level analyses, five types of conditions were defined: (i) the outcome was non-optimal and participants focused on collected gains (Nopt_GF, *M* = 7.4 trials, *SD* = 2), (ii) the outcome was non-optimal and participants focused on missed chances (Nopt_MF, *M* = 22.4 trials, *SD* = 2.8),(iii) the outcome was optimal and participants focused on collected gains (Opt_GF, *M* = 8.2 trials, *SD* = 2.4), (iv) the outcome was optimal and participants focused on missed chances (Opt_MF, *M* = 20.7 trials, *SD* = 3.9), and (vi) trials in which participants unpacked the devil (Devil, *M* = 31.2 trials, *SD* = 5.1). All of conditions were time-locked to the presentation of the outcome of final decision with a duration of 3 s, convolved with a canonical hemodynamic response function (HRF). Additional covariates of no interest were created for decision-making phase (also convolved with HRF) and movement-related variance. High pass temporal filtering with a cutoff of 128 s was also applied in the model. The five first level contrast images (Opt_GF, Opt_MF, Nopt_GF, Nopt_MF and Devil) from each participant were then analyzed at the second level employing a random-effects model (flexible factorial design in SPM12).

To test our hypothesis, we first conducted region of interest (ROI) analyses to examine the affect of attentional deployment (Context: GF vs. MF) on regret on neural level. Previous researches have revealed that the brain region of striatum was associated with regret processing, so our first ROI was striatum (left putamen, MNI −22 8 8; right putamen, MNI 24 8 4)^3^,^14^,^15^. Moreover, according to previous findings of the involvement of right middle frontal gyrus (MFG) in attentional deployment processing, our second ROI was right MFG (MNI 34 49 19)^10^. Additionally, regions of pregenual anterior cingulate cortex (pgACC, MNI −4 44 4) and medial prefrontal cortex (mPFC, MNI −4 58 10) were found specially in our previous research by adopting the similar experimental paradigm^15^. These regions were also defined as ROIs in the current study. Finally, five ROIs were defined as spheres with a radius of 8 mm centered at MNI coordinates −22/8/8 (left putamen), 24/8/4 (right putamen), 34/49/19 (right MFG), −4/44/4 (pgACC) and −4/58/10 (mPFC) by using MarsBar toolbox in SPM12. Parameter estimates of signal intensity were extracted from these ROIs and subjected to a repeated measures analyses of variance (ANOVA) with Outcome (Nopt vs. Opt) and Context (GF vs. MF) as within-subjects variables. Multiple comparisons correction was performed using a false discovery rate (FDR) to correct for the number of tested ROIs using the Benjamini–Hochberg (BH) method^37^. We followed up our ROI analyses with whole-brain regression analyses. Additionally, activations of brain regions, like striatum and so on, were found in Nopt - Devil contrast in our previous research. In the current study, the same contrast was also conducted to verify our previous findings. All clusters in whole-brain regression analyses survived family wise error (FWE) correction (p < .05) for multiple comparisons at the peak level corrected.

## Additional Information

**How to cite this article**: Liu, Z. *et al*. Attentional deployment impacts neural response to regret. *Sci. Rep.* **7**, 41374; doi: 10.1038/srep41374 (2017).

**Publisher's note:** Springer Nature remains neutral with regard to jurisdictional claims in published maps and institutional affiliations.

## Figures and Tables

**Table 1:** Conjunction analyses of (Opt - Nopt) and (GF - MF) contrasts.

	Region	Peak Activation			*t* Value	Voxels
		X	Y	Z		
L	Putamen	−16	14	−2	6.87	447
L	*Thalamus*	−6	−6	8	6.36	
R	*Putamen*	18	10	2	5.71	
R	*Thalamus*	4	−12	8	5.42	
R	pgACC	8	36	12	5.47	41
L	pgACC	−6	28	16	5.62	24

Note. Coordinates (mm) are in MNI space. L = left hemisphere; R = right hemisphere. All of the clusters survived FWE correction (p < 0.05) for multiple

comparisons at the peak level corrected.

**Table 2:** (Opt-Nopt) contrast exclusively masked by (GF-MF) contrast.

	Region	Peak Activation			*t* Value	Voxels
		X	Y	Z		
L	mPFC	−4	54	2	7.49	2020
L	*dmPFC*	−6	58	24	6.7	
L	*Mid orbital gyrus*	−2	46	−12	6.35	
R	*pgACC*	8	46	6	5.41	
R	Cerebelum	40	−72	−36	6.98	128
L	Middle cingulate cortex	−12	−46	32	5.86	79
L	Inferior temporal gyrus	−54	−24	−20	5.9	52

Note. Coordinates (mm) are in MNI space. L = left hemisphere; R = right hemisphere. The mask image was thresholded at P < 0.05 uncorrected. All of the clusters survived FWE correction (p < 0.05) for multiple comparisons at the peak level corrected.

**Table 3:** (GF - MF) contrast exclusively masked by (Opt - Nopt) contrast.

	Region	Peak Activation			*t* Value	Voxels
		X	Y	Z		
R	Supplementary motor area	2	10	52	9.97	11419
R	*Middle cingulate cortex*	6	18	42	9.62	
R	*SFG*	26	8	58	8.6	
R	*Insula lobe*	46	14	0	5.53	
R	Lingual gyrus	24	−84	−6	10.22	2107
R	IPL	34	−50	50	9.83	1999
R	MFG	36	52	12	6.88	488
L	Cerebellum	−22	−68	−48	7.89	240
L	Insula lobe	−28	24	8	7.69	172
L	Rolandic operculum	−50	0	4	6.25	95
R	Superior temporal gyrus	68	−36	20	5.92	55

Note. Coordinates (mm) are in MNI space. L = left hemisphere; R = right hemisphere. The mask image was thresholded at P < 0.05 uncorrected. All of the clusters survived FWE correction (p < 0.05) for multiple comparisons at the peak level corrected.

**Table 4:** The effect of Non-optimum (Nopt) and Devil outcomes.

	Region	Peak Activation			*t* Value	Voxels
		X	Y	Z		
***Nopt - Devil***						
L	Fusiform Gyrus	−32	−62	−10	14.6	19940
R	*Superior parietal lobule*	30	−56	48	14.33	
R	Inferior frontal gyrus	50	12	28	13.82	7502
R	*Putamen*	20	12	2	7.98	
R	*Middle orbital gyrus*	48	54	−2	6.43	
L	Insula lobe	−30	24	0	11.04	4275
L	*Putamen*	−18	10	0	7.15	
L	*Inferior frontal gyrus*	−48	8	4	6.38	
L	*Pallidum*	−18	−2	6	5.92	
R	Parahippocampal	24	−30	−10	5.22	62
***Devil - Nopt***						
	no regions					

Note. Coordinates (mm) are in MNI space. L = left hemisphere; R = right hemisphere. All of the clusters survived FWE correction (p < 0.05) for multiple comparisons at the peak level corrected.
